# Discrepancies in self-reported and measured anthropometric measurements and indices among older Australians: prevalence and correlates

**DOI:** 10.1186/s12889-022-14326-y

**Published:** 2022-10-17

**Authors:** Jane M. Fry, Jeromey B. Temple

**Affiliations:** Melbourne School of Population and Global Health, University of Melbourne, VIC, 3010 Melbourne, VIC Australia

**Keywords:** Height, Weight, Body Mass Index (BMI), Misperception, Self-report, Older people

## Abstract

**Background:**

Anthropometric measurements and indices such as weight, height and Body Mass Index (BMI) are often used to assess overall health and nutritional status. Clinicians and epidemiologists often rely on self-reported weight and height to measure BMI. Differences between self-reported and measured weight and height can lead to differences between self-reported and measured BMI, biasing relative risks of diseases associated with differential BMI.

**Methods:**

Applying regression analysis to a large nationally representative survey data with contemporaneous self-reports and measurements on 3412 individuals aged 65 or over, we provided estimates of the difference between self-reports and measurements of weight, height and BMI for older Australians, analysing demographic, socioeconomic and health correlates of estimated differences.

**Results:**

We found both males and females underestimated weight, overestimated height and underestimated BMI and there was some evidence these differences increased with age. There was also evidence that these differences were associated with high levels of education and household composition.

**Conclusion:**

Although average differences were small, for many individuals the differences may be significant, indicating measurements should be taken in clinically focused research and practice. This is important as systematic underestimation of BMI in older adults can have implications for estimating the size of populations at risk of many health conditions, including diabetes, hypertension and functional limitations.

**Supplementary Information:**

The online version contains supplementary material available at 10.1186/s12889-022-14326-y.

## Introduction

Anthropometric measurements and indices such as weight, height and Body Mass Index (BMI, defined as weight in kilograms divided by height in metres squared) are non-invasive, easily applied and provide an indicator of overall health and nutritional status. In particular, these measurements can indicate obesity or overweight status, malnutrition, vertebral compression, loss of muscle tone and postural slump [1].Overweight and obesity can have major health consequences, such as increased arthritis, cardiovascular diseases, diabetes, hypertension, musculoskeletal disorders (including osteoarthritis) and some cancers — conditions particularly relevant to later life [2, 3]. Malnutrition affects body composition in terms of reduced fat-free mass and particularly affects older people, with an estimated 23–39% of older adults admitted to hospital being malnourished and 43–46% at risk of malnutrition [5]. Malnutrition increases with dependency and care needs, with the lowest prevalence among older adults in the community and the highest prevalence among older adults in hospitals and rehabilitation facilities [6].

Differences between self-reported and measured weight and height can lead to differences between self-reported and measured BMI, biasing relative risks of diseases associated with an increasing BMI [7]. Clinicians and epidemiologists often rely on self-reported weight and height to measure the BMI as a comparable measure of obesity and overweight across the population [7, 8]. However, if concordance between self-reported and measured weight and height is low, BMI estimates may be biased and this affects estimates of prevalence of overweight, obesity and malnutrition at a macro level and weight-related attitudes and behaviours and treatment options at the micro level [9].

The literature relating to bias in reported anthropometric measurements and indices among older people is limited, despite evidence showing differences between self-reported and measured weight, height and BMI increases with age [1]. Studies focusing on older people are important due to greater risks of adverse health conditions associated with being overweight and/or having reduced stature. Although being overweight is important across all age groups, when combined with frailty associated with ageing, its importance is amplified. Moreover, further studies among older people are warranted given the rapid increase in inexorable population ageing worldwide. The World Health Organisation estimates that between 2015 and 2050, the world population aged 65 and over will double.^1^ In Australia, despite older people accounting for a disproportionate share of COVID-19 deaths, the proportion of the population aged 65 and over is projected to grow to about 1 in 5 people by 2041 [10].

Within this context, we sought to evaluate the concordance between self-reported and recorded (actual) weight, height and BMI in a nationally representative sample of older Australians.

## Background and literature review

Bias in self-reported data may be unique to each population [11], so we considered studies from various countries. One US study of people aged aged 60 or over found significant differences between self-reported and measured weight, height and BMI [1]. Self-reported weight was 0.51 kg higher than measured weight for men but 0.56 kg lower for women; self-reported height was higher than measured height by an average of 2.7 cm for men and 2.5 cm for women; and self-reported BMI was lower than measured BMI (0.64 kg/m^2^ for men and − 1.05 kg/m^2^ for women). The authors also found the difference in weight was also positive and increased with age for males. For females, it also increased with age and the difference between self-reported and measured height was positive and increased with age for both sexes.

Analysis of the same dataset showed the lowest level of education was associated with higher overestimation of weight by males and less underestimation of weight by females [12]. Similar findings were associated with poverty for both sexes and with self-assessed health for males. In terms of height, overestimation by males and females was higher for those with less education and in the lowest poverty group. Living alone was also associated with greater overestimation of height by females. Poor self-assessed health was associated with increased overreporting of height by both sexes. The extent of underreporting of BMI increased with age, and with self-assessed health for men. Poor cognition, which has been associated with psychological distress [13], was also associated with significant *over*estimation of weight for females and overestimating height for both sexes.

Another s US study showed self-reported weight among individuals aged 50–65 was less than measured weight for both males (-1.51 kg) and females (-0.93 kg). Self-reported height was greater than measured height for males (0.59 cm) and females (0.29 cm). Self-reported BMI was also lower than measured BMI (-0.66 kg/m^2^ for males and − 0.45 kg/m^2^ for females) [14]. However, despite a similar result for height, in analysing US data for individuals aged 65 years or over, Maclean & Kessler (2015) found the underestimate of weight and BMI was larger for females than for males [15]. Among 87 adults aged 60 years or over in Brasilia, both males and females showed self-reports were higher than measurements for weight and height, with larger differences for men than women [16]. These differences were unrelated to education levels or other socioeconomic indicators.

There are also relevant studies for Europe. Niedźwiedzka et al. (2015) found similar results for older Poles, although their sample was limited to 102 individuals [11]. For weight, height and BMI the correlations between self-reports and measurements were very high at r = 0.94 or higher. In Sweden, self-reported weight among 595 older people (aged 45 or over) was lower than measured weight (by 1 kg on average), self-reported height was higher than measured height (0.9 cm) and for BMI self-reports were lower than measurements (-0.6 kg/m^2^) [17]. Gunnell et al. (2000) studied 257 individuals aged 56–78 years in Britain and found for both sexes weight was underestimated, and height and BMI overestimated (with larger differences for men on all three metrics) [18]. Age was significantly correlated with height (larger differences with age) and low socioeconomic status with weight (larger differences).

A Japanese study of persons aged 65–89 years showed no significant difference in self-reported and measured weight for males and females, but self-reported height exceeded measured height by 2 cm for males and 3 cm for females and self-reported BMI was lower than measured BMI (0.4 kg/m^2^ for males and 1.0 kg/m^2^ for females) [19]. An earlier Japanese study found older males and females underestimated weight by about 1 kg, overestimated height by about 1 cm and underestimated BMI by 0.7–0.8 kg/m^2^ [20]. Underestimates of weight increased with age for males but declined for females. Overestimates of height increased with age for both sexes and underestimates of BMI increased with age.

A multi-country study of 14,650 individuals aged 50 years or over in China, India, Russia and South Africa found self-reported weight was lower than measured weight in India, Russia and South Africa (typically by 1 kg or less) but higher for China (up to 7.6 kg for those aged 80 years or over) [21]. In India and South Africa, self-reported height was lower than measured height by 2–6 cm, but the reverse was true for China and Russia (up to 3 cm higher). Self-reported BMI was lower than measured BMI for Russia (approx. 0.4 kg/m^2^) but higher in the other three countries (up to 2.4 kg/m^2^) (Ng 2019). Interestingly, in each country, males and females showed the same direction of ‘bias’.

More closely related to our study, Australian estimates for New South Wales based on 608 individuals aged 45 or over showed self-reported weight was lower than measured weight by 1.68 kg for males and 1.02 kg for females. Self-reported height was higher than measured height by 1.24 cm for males and 0.59 cm for females and self-reported BMI was lower than measured BMI (0.9 kg/m^2^ and 0.6 kg/m^2^ for males and females respectively). Correlations between contemporaneous self-reports and measurements were very high at 0.95 or higher for the full sample [22]. A study of self-perceived BMI showed among individuals aged 50 or over in Australia, perception of being overweight decreased with age and perception of being underweight increased with age [23].

Analysing different age groups among older people is important as there may be differences between self-reports and measurements, although, based on a relatively small sample, Dahl et al. (2010) found a very small increase for BMI differences with ageing in Sweden [17]. Data for the US showed increased overreporting of weight by age in males and reduced underreporting of weight for females and increased overreporting of height for both sexes [12]. In New South Wales, for males the positive difference between self-reported and measured height increased with age [22]. Among older people, measured height may exceed self-reported height if individuals report height recalled from early adulthood, being unaware of changes in stature due to postural problems [17], reductions in muscle mass and bone density or declines in cognition [15]. Osteoporosis in older women reduces their measured height. However, there is evidence that a diagnosis of osteoporosis is associated with increased accuracy in self-reported height — perhaps due to access to physicians, better self-monitoring of body changes or due to awareness of the condition [7].

In using self-report data for older Australians, it is therefore important to analyse differences from measurements using large sample, nationally representative data. Our contribution was to provide estimates of the difference between self-reports and measurements of weight, height and BMI for older Australians. Moreover, we analysed demographic, socioeconomic and health determinants of such differences. In contrast to many studies, we used a nationally representative large sample. A strength of our data lay in contemporaneous self-reports and measurements, meaning there was no time lapse in which to gain weight and therefore alter measured weight and BMI.

## Methods

Data for this study were from the National Health Survey (NHS) 2017-18 conducted by the Australian Bureau of Statistics between July 2017 and June 2018. The sample was a stratified, multistage cluster sample of households, covering all states and territories and captured 21,315 respondents in 16,384 private dwellings in urban, rural and remote areas of Australia, with a response rate of 76% [24, 25]. For each selected household, one adult and one child aged 0–17 was selected as a survey respondent. Data were obtained through a face-to-face interview.

NHS interviewers were selected from a pool of trained interviewers with experience on other ABS household surveys. Interviewers undertook additional training and study exercises specific to the NHS. Training focused on survey concepts, definitions, procedures and equipment use to ensure all interviewers adopted a standard approach to data collection. Specifically, written instructions on how to use each piece of equipment and pack the equipment was sent to interviewers and was available online. Additionally, a demonstration video was made available to Interviewers at training and via the online database for future reference. This also highlighted the importance of Occupational Health and Safety and interviewer responsibilities when using the equipment. A Face-To-Face training was provided to Interviewers which involved watching a video; an equipment quiz; hands-on practice with the equipment; group discussion of OHS issues, risks and safety relating to the use and manual handling of the equipment.

Interviewers used brand new digital scales to measure weight (maximum 200 kg). Weight was recorded in kilos to one decimal point. Weight measurements were only taken once. A brand new stadiometer was used to measure height (maximum 210 cm). Height measurements were recorded in centimetres to one decimal point. As the stadiometers and scales were brand new, no testing or calibrating was done. However, an additional height measure was taken to analyse variation for quality assurance. Interviewers encouraged respondents to remove shoes and heavy clothing before taking measurements and all respondents were measured using the same equipment. The ABS used the WHO measurement protocol for weight and height [26].

Questions in the NHS focused on health status and health-related aspects of lifestyle, including health conditions, smoking, alcohol consumption, diet, physical activity, physical measurements and medication use. Given the multi-stage sampling technique and non-random selection of respondents by the Australian Bureau of Statistics, adjustments were necessary to account for the complex survey design in order to obtain correct variance estimates for the population. Our adjustments used the unstratified delete-one jackknife method with 60 replicate weights from the data file.

Our sample comprised 4187 individuals aged 65 or over, of which 3466 had self-reported and measured height, weight and BMI measurements. Respondents were asked their weight and height (without shoes), and BMI was calculated based on these responses. Subsequently, measurements (without shoes and in light clothing) were taken using digital scales and a stadiometer. In cleaning the data, we removed individuals with self-reports or measurements greater than 4 standard deviations from the mean, leaving 3412 individuals with plausible anthropometric data. We removed these few individuals as their range of discrepancies in the anthropometric variables were more than double the ranges for the individuals we kept. For example, some individuals had implausibly large differences in weight of up to 90 kg, height of up to 62 cm and BMI of up to 49 kg/m^2^. By dropping these individuals, we were trimming the data. We did this on the premise that including such extreme observations may have distorted means and inflated the estimated standard errors [27, 28].^2^ This approach is common in the literature [17, 29, 30]. Our empirical work identified 6 chronic conditions with high morbidity/prevalence for which we examined associations with discrepancies in weight, height and BMI, namely arthritis, cardiovascular disease, diabetes, hypertension, musculoskeletal disease and cancer. Other studies have noted the importance of these chronic conditions for older Australians [31].

Having described the data, our analysis began with plots of self-reports against measurements and Bland-Altman plots of differences between the two [32] and continued with estimated differences by age and sex. Finally, we estimated linear regressions of the form:

1 $${\left(SR-M\right)}_{i}=X\beta +{u}_{i}$$

where (SR-M) is the difference between self-reported and measured weight, height or BMI (outcomes), X is a set of demographic, socioeconomic and health variables and u is the usual error term. All models used robust standard errors [33].

## Results

### Descriptive statistics

In our population-weighted sample, measured weight was approximately 78 kg and on average was higher than self-reported weight by 1 kg (Table 1). Measured height was 164 cm and was lower than self-reported height by 3.6 cm. BMI was classified into 6 categories: underweight (< 18.5), normal (18.5–24.9), overweight (25–29.9), obese I (30.0–34.9), obese II (35–39.9) and obese III (40 and above). The average measured BMI was at the high end of overweight at 28.9 kg/m^2^ and was higher than self-reported BMI at 27.3 kg/m^2^. However, there was substantial variation in these differences with a range of -33–31 kg, -23–30 cm and − 13–10 kg/m^2^. The largest age group was aged 65–69 years (33%). Females accounted for 50% of the population. Education levels were relatively low with almost half of the sample having only schooling below Year 12. Most individuals were born in Australia (63%) with the remainder split between mainly English-speaking (16%) and non-English speaking countries (21%). Most individuals were retired and thus not in the labour force, although 16% were employed. A large proportion were pensioners (58%). The overwhelming majority mainly spoke English (91%). Approximately 62% lived with a disability and self-assessed health overall was predominantly good to excellent (76%). Relative incomes were moderate with 73% in the second or third quintile for the Australian population. Most households had 2 adult occupants (60%). Most individuals lived in major cities (66%), although a substantial number lived in inner regional areas or more remote areas of Australia. Consistent with national coverage of the sample, all states and territories were represented in the data.

**Table 1:** Means by measure (SR – M), population weighted

	Sample n	Weight	Height	BMI
		SR–M Mean	SR–M Mean	SR–M Mean
*Age*				
65–69	1130	-0.882	3.279	-1.428
70–74	996	-1.195	3.089	-1.504
75–79	637	-0.732	4.196	-1.680
80–84	397	-1.906	3.765	-2.037
85+	253	0.112	5.615	-1.737
*Sex*				
Male	1706	-0.722	4.045	-1.556
Female	1706	-1.257	3.216	-1.629
*Education*				
University	623	-1.646	3.730	-1.799
Diplomas and certificates	914	-1.063	3.513	-1.558
Year 12	297	-0.824	3.416	-1.424
Less than Year 12	1579	-0.707	3.705	-1.560
*Country of birth*				
Australia	2143	-0.670	3.988	-1.600
Mainly English Speaking	559	-0.638	3.601	-1.427
Other	709	-2.213	2.596	-1.700
*Labour Force Status*				
Employed	529	-0.655	3.930	-1.526
Not employed	2883	-1.052	3.576	-1.605
*Pensioner status*				
No	1429	-1.211	3.509	-1.582
Yes	1983	-0.806	3.734	-1.601
*English proficiency*				
Mainly English	3098	-0.828	3.762	-1.574
Well	158	-1.689	2.527	-1.508
Not well	155	-3.547	2.152	-2.055
*Disability*				
No disability	1287	-0.920	3.401	-1.464
Disability	2125	-1.030	3.776	-1.672
*Self assessed health*				
Excellent	417	-1.016	3.516	-1.524
Very good	1047	-0.967	3.658	-1.555
Good	1137	-0.939	3.724	-1.617
Fair	566	-1.125	3.506	-1.661
Poor	246	-0.939	3.605	-1.604
*Income quintile*				
Lowest	390	-1.036	3.279	-1.498
2	1601	-0.894	3.659	-1.615
3	693	-0.923	3.912	-1.626
4	276	-1.138	3.627	-1.548
Highest	180	-1.433	3.493	-1.654
*Adults in household*				
1	989	-1.063	3.248	-1.482
2	2046	-0.858	3.788	-1.587
3 or more	377	-1.521	3.793	-1.923
*Area*				
Major city	2244	-0.990	3.716	-1.614
Inner regional	776	-0.926	3.478	-1.511
Other	392	-1.104	3.457	-1.627
*Number of conditions*				
0	16	-1.223	5.140	-2.012
1	108	-0.501	3.327	-1.279
2	207	-1.309	2.577	-1.276
3	305	-1.163	3.281	-1.516
4	367	-1.198	3.636	-1.633
5 or more	2,409	-0.927	3.769	-1.633
*Specific conditions*				
Arthritis	1071	-1.027	3.533	-1.615
Cardiovascular disease	688	-1.094	3.676	-1.631
Diabetes	524	0.012	3.581	-1.274
Hypertension	1495	-0.907	3.791	-1.671
Musculoskeletal disorders	1954	-0.756	3.854	-1.611
Cancer	1084	-0.843	3.930	-1.619

**Fig. 1:**
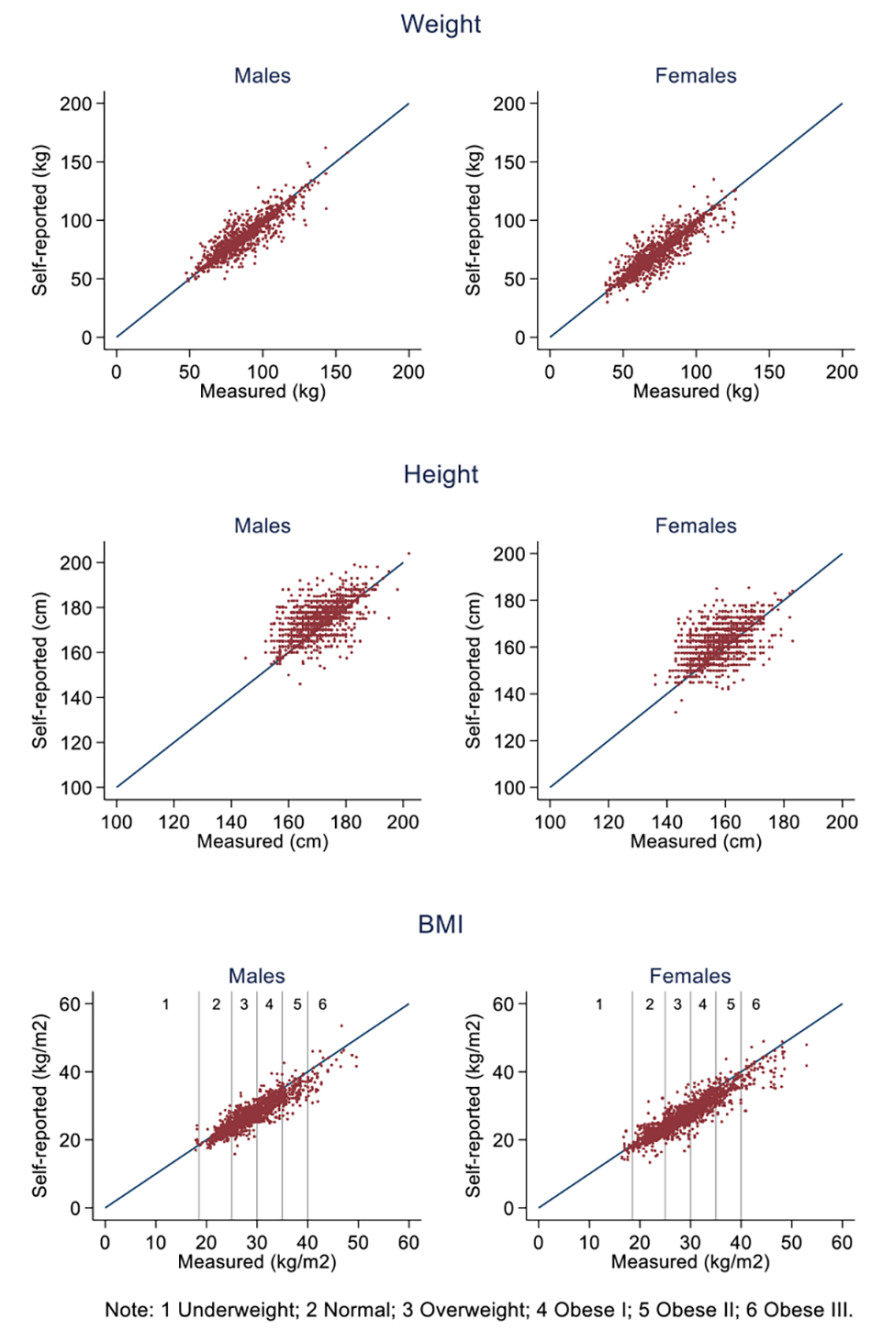
Self-reports and measurements, unweighted

Figure 1 shows self-reported against measured weight, height and BMI for males and females. In each chart, the 45 degree line shows equality between self-reports and measurements. For most individuals in the sample, there was close agreement between the two measures for weight and BMI. However, the agreement was slightly less for height, with males and females overestimating height. Measured BMI fell mostly between the normal range and obese I for both males and females.

**Fig. 2:**
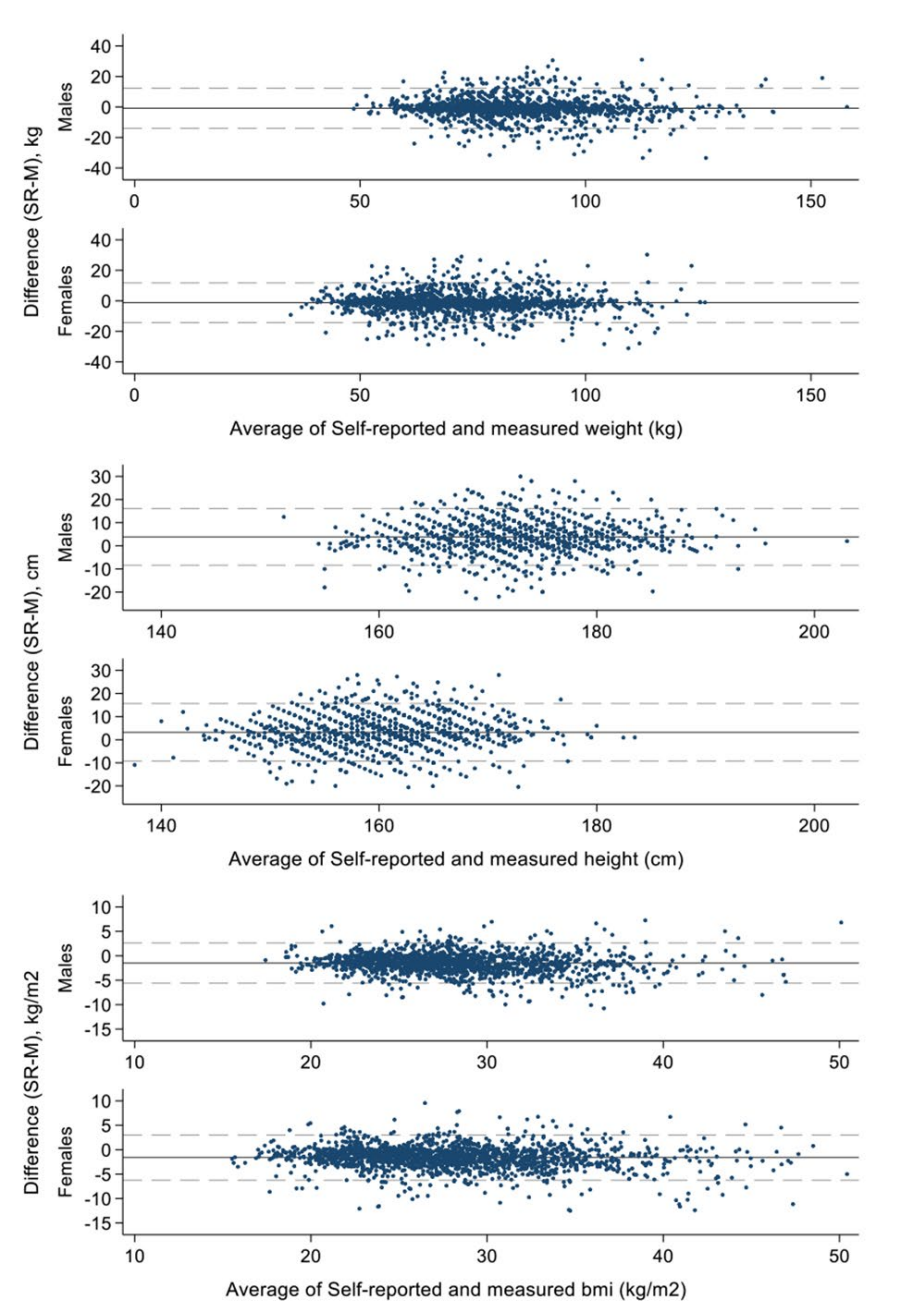
Bland-Altman plots for weight, height and BMI. The solid line indicates mean difference and the dashed lines indicate the limits of agreement (mean ± 1.96SD), unweighted

Bland-Altman plots (Fig. 2) show the difference between self-reports and measurements plotted against the average of self-reports and measurements for males and females. These plots reveal that males tended to be heavier than females, but that females showed larger differences between self-reports and measurements (a greater number of individuals had significant differences between self-reported and measured weight). Males also tended to be taller than females but had similar propensity to under or over estimate their height. Despite differences in weight and height, BMI measurements and differences from self-reports were similar between the two sexes.

In Table 2 we explore differences between self-reports and measurements by age and sex. Apart from height of individuals aged 65–69, there were no significant differences between the sexes in self-reported minus measured weight, height or BMI. Both males and females underestimated weight, overestimated height and underestimated BMI.

**Table 2:** Self-reported – Measured outcomes by age and sex, population weighted

	Self-Report – Measured										
	Males					Females					
	Weight (kg)										
Age	Population	mean	se(mean)	LL	UL		Population	mean	se(mean)	LL	UL
65–69	507,678	-0.589	0.272	-1.123	-0.055		462,574	-1.203	0.360	-1.908	-0.498
70–74	437,458	-0.897	0.367	-1.616	-0.178		433,682	-1.496	0.307	-2.098	-0.893
75–79	272,861	-0.396	0.406	-1.193	0.400		294,096	-1.043	0.372	-1.772	-0.315
80–84	166,816	-1.890	0.698	-3.257	-0.522		180,629	-1.921	0.564	-3.027	-0.816
85+	114,854	0.272	0.648	-0.997	1.541		112,860	-0.050	0.495	-1.020	0.920
total	1,499,666	-0.722	0.178	-1.072	-0.373		1,483,841	-1.257	0.166	-1.582	-0.932
	Height (cm)										
65–69	507,678	3.981	0.251	3.490	4.472		462,574	2.510	0.384	1.756	3.263
70–74	437,458	3.408	0.347	2.729	4.087		433,682	2.768	0.368	2.046	3.490
75–79	272,861	4.683	0.549	3.608	5.758		294,096	3.745	0.360	3.040	4.450
80–84	166,816	3.381	0.487	2.426	4.336		180,629	4.119	0.583	2.975	5.262
85+	114,854	6.206	0.619	4.992	7.420		112,860	5.015	0.548	3.940	6.089
total	1,499,666	4.045	0.172	3.708	4.382		1,483,841	3.216	0.183	2.858	3.575
	BMI										
65–69	507,678	-1.503	0.071	-1.642	-1.363		462,574	-1.346	0.110	-1.563	-1.130
70–74	437,458	-1.414	0.112	-1.632	-1.195		433,682	-1.594	0.113	-1.816	-1.372
75–79	272,861	-1.630	0.130	-1.885	-1.374		294,096	-1.726	0.155	-2.029	-1.423
80–84	166,816	-1.749	0.204	-2.149	-1.350		180,629	-2.302	0.205	-2.704	-1.901
85+	114,854	-1.877	0.301	-2.466	-1.288		112,860	-1.595	0.207	-2.001	-1.188
total	1,499,666	-1.556	0.054	-1.662	-1.449		1,483,841	-1.629	0.053	-1.734	-1.525

Note: LL and UL are 95% confidence interval limits

Although not significantly different from younger age groups, there were indications that some older people in the 80–84 years age bracket may have had larger underestimates of weight and some in the 85 years or over may have had larger overestimates of height, however, this may become significant with larger samples.

Table 3 shows Pearson correlations between self-reports and measurements by age, sex and outcome. In all cases, the correlations were positive and large, indicating self-reports and measurements tended to move in the same direction. These results indicated correspondence between self-reports and measurements broadly were higher for weight and BMI than for height and declined with age.

**Table 3:** Pearson correlations between self-reports and measurements by age and sex, population weighted

	Males			Females		
	Weight	Height	BMI	Weight	Height	BMI
65–69	0.917	0.708	0.922	0.916	0.555	0.936
70–74	0.901	0.606	0.906	0.902	0.628	0.914
75–79	0.876	0.564	0.869	0.895	0.517	0.913
80–84	0.879	0.575	0.910	0.884	0.496	0.878
85+	0.797	0.490	0.819	0.822	0.508	0.851
Total	0.903	0.637	0.903	0.904	0.574	0.915

Noting that there only appeared to be a significant difference in height between males and females aged 65–69 years, we estimated our linear regression models on the pooled sample (both sexes) for each dependent variable — indicating self-report minus measurement variables for weight, height and BMI respectively (Table 4). Each model included all of our demographic and socioeconomic variables (and States). Complementary research — particularly in relation to obesity — has considered a selection of common health conditions, so we included arthritis, cardiovascular disease, diabetes, hypertension, musculoskeletal disorders and cancer in our analysis as potentially important correlates.

**Table 4:** Linear regression results for self-reported – measured outcomes

	Weight	Height	BMI		Weight	Height	BMI		Weight	Height	BMI
*Age (ref: 65–69)*				*Pensioner status (ref: non-pensioner)*				*Adults in household (ref: one)*			
70–74	-0.341	-0.140	-0.101	Pensioner	0.495	0.349	0.0383	Two adults	0.291	0.770***	-0.175
	(0.320)	(0.361)	(0.101)		(0.378)	(0.350)	(0.145)		(0.310)	(0.274)	(0.115)
75–79	0.093	0.955**	-0.268*	*English proficiency (ref: not well)*				Three adults	0.093	1.169**	-0.453**
	(0.374)	(0.446)	(0.137)	Mainly English speaking	1.600	1.237	0.115		(0.620)	(0.550)	(0.221)
80–84	-0.814	0.623	-0.563***		(0.957)	(0.829)	(0.346)	*Area (ref: Major city)*			
	(0.568)	(0.548)	(0.199)	Speaks English well	2.076**	0.520	0.540	Inner regional	-0.473	-0.688**	0.068
85+	1.008	2.496***	-0.342		(1.018)	(0.863)	(0.423)		(0.360)	(0.341)	(0.104)
	(0.694)	(0.578)	(0.247)	*Disability (ref: none)*				Other region	-0.555	-0.531	-0.073
*Sex (ref: male)*				Disability	-0.473	-0.089	-0.156		(0.636)	(0.459)	(0.182)
Female	-0.746**	-0.939***	-0.101		(0.370)	(0.289)	(0.125)	Number of conditions	0.012	-0.041	0.021
	(0.309)	(0.319)	(0.090)	*Self assessed health (ref: poor)*					(0.054)	(0.041)	(0.020)
Education (ref: <year12)				Excellent	0.255	-0.062	0.116	*Specific conditions*			
University	-1.015**	-0.010	-0.289**		(0.770)	(0.801)	(0.231)	Arthritis	-0.182	-0.291	0.020
	(0.409)	(0.357)	(0.138)	Very good	0.136	-0.229	0.151		(0.312)	(0.305)	(0.116)
Diplomas and Certificates	-0.434	-0.240	-0.057		(0.688)	(0.690)	(0.172)	Cardiovascular disease	-0.387	-0.295	-0.019
	(0.347)	(0.324)	(0.139)	Good	0.101	-0.040	0.033		(0.440)	(0.381)	(0.117)
Year 12	-0.067	-0.155	0.060		(0.680)	(0.670)	(0.184)	Diabetes	1.040**	-0.179	0.371**
	(0.522)	(0.429)	(0.169)	Fair	-0.240	-0.353	-0.036		(0.439)	(0.410)	(0.150)
*Country of Birth (ref: other)*					(0.810)	(0.785)	(0.244)	Hypertension	0.034	0.404	-0.195**
Born in Australia	1.478***	1.302**	0.092	*Income quintile (ref: Bottom quintile)*					(0.248)	(0.303)	(0.094)
	(0.512)	(0.524)	(0.165)	Second quintile income	-0.143	0.217	-0.151	Musculoskeletal disorders	0.817**	0.768**	0.0140
Mainly English speaking countries	1.448***	0.547	0.347**		(0.490)	(0.479)	(0.196)		(0.360)	(0.344)	(0.119)
	(0.541)	(0.500)	(0.168)	Third quintile	-0.189	0.417	-0.211	Cancer	-0.053	0.013	-0.008
*Labour force status (ref: not employed)*					(0.562)	(0.529)	(0.186)		(0.291)	(0.300)	(0.103)
Employed	0.445	0.628	-0.053	Fourth quintile	-0.325	-0.000	-0.089	Constant	-3.799***	0.922	-1.504***
	(0.501)	(0.460)	(0.179)		(0.798)	(0.727)	(0.226)		(1.362)	(1.176)	(0.413)
				Top quintile	-0.614	-0.237	-0.182	States	yes	yes	yes
					(0.676)	(0.754)	(0.299)				

Note: Dependent variables are Self-report — Measured. Robust standard errors in parentheses. Regressions included all listed variables. n = 3139. R^2^: 0.037 (weight), 0.039 (height), 0.028 (BMI). *** p < 0.01, ** p < 0.05, * p < 0.1

Our data contained information on over 100 specific health conditions and we investigated weight, height and BMI differences for the most prevalent conditions and comorbid conditions. Analysis showed considerable homogeneity as there were no significant differences in means for any of the three variables. A variety of other health-related variables were also examined (short/long-term alcohol consumption, fruit and vegetable consumption, exercise and physical activity, smoking status, and Kessler 10 distress category) and no significant effects were found. We also considered numbers of conditions as a proxy for general health and found no significant differences. These results are presented in the appendix.

We found no significant relationship of underestimates of weight with age. Females underestimated weight by 0.75 kg more than males. Those with university education underestimated weight by 1.0 kg more than their least educated counterparts. Compared to those born in non-English speaking countries, those born in Australia or mainly English speaking countries underestimated weight by 1.4 kg more. Those who mainly spoke English underestimated weight by 1.6 kg less than did those who did not speak English well and those who spoke another language and spoke English well underestimated by 2.1 kg less. Individuals with diabetes underestimated weight by 1.0 kg and those with musculoskeletal disorders underestimated by 0.8 kg less.

Overestimates of height broadly increased with age and those aged 85 years or over had overestimates that were 2.5 cm more than for those aged 65–69 years. Overestimates by females were 0.9 cm smaller than those for males. Being born in Australia increased overestimates of height by 1.3 cm over that reported by individuals from non-English speaking countries. Those living in households with two or more adults overestimated by more than those in single person households (0.77 cm for two adults and 1.17 cm for three or more adults). Individuals living in inner regional areas overestimated height by 0.7 cm less than those living in metropolitan areas. Of all the specified health conditions, only musculoskeletal disorders were associated with significant differences in height (overestimate by 0.8 cm more).

On average, BMI was underestimated. The extent of underestimation increased with age until age 85 or over. Those aged 75–79 years underestimated BMI by 0.3 kg/m^2^ compared to individuals aged 65–69 years. Similarly, 80–84 year olds underestimated by 0.6 kg/m^2^. University education was also associated with a greater degree of underestimation of BMI of 0.3 kg/m^2^ compared to those with less than Year 12 education. Those born in mainly English speaking countries underestimated by 0.3 kg/m^2^ less than those born in non-English speaking countries. Living in a household with three or more adults was associated with a 0.5 kg/m^2^ larger underestimate of BMI. Having diabetes was associated with a 0.4 kg/m^2^ smaller underestimate of BMI and hypertension a 0.2 kg/m^2^ larger underestimate.

## Discussion

Our results on weight for females were consistent with those in the literature [1, 14] as older females underestimated weight. Consistent with Hodge et al. (2020) and Yong & Saito (2012) [14, 20] we found males also underestimated weight. Our results were consistent with those of several studies for NSW [22], for Britain [18], for India, Russia and South Africa [21] and for Japan [20]. Compared to the least educated, the most educated had greater underestimation, consistent with results for India and Russia [21], Catalonia [34], the US [15, 35] and Poland [36]. This is not what would be expected if education is associated with health literacy and could reflect greater time since last measurements were taken. There was less underreporting from those born in Australia compared to born in non-English speaking countries but not compared to those born in mainly English speaking countries. Our result echoed that of Howard et al. (2008) who found weight underestimation was greater for those born in Eastern and Western Europe than those born in Australia and that there was no significant difference in underestimation between individuals born in the UK or Ireland compared to those born in Australia [37]. This could be consistent with greater healthcare utilisation (or the significantly larger number of health conditions that Australian-born individuals in our data suffered) for Australian-born individuals leading to more clinical attention on weight. It could also be due to ‘sociocultural factors that drive the standards of desirable body weight within cultures, which in turn drive the lifestyles that people lead’ [37]. Membership of the manual social class in Britain has been associated with differences in self-reported and measured weight (larger differences) [18]. However, as a proxy for socioeconomic status, we found no differences by levels of income. This may have been due to offsetting effects of males and females [12]. An underestimate of weight by individuals with diabetes or musculoskeletal disorders is consistent with, Gil & Mora (2011) who found Catalans underestimated weight by significantly less if they had (unspecified) chronic diseases [34]. However, US data showed males overestimated weight by more if they had osteoporosis [12].

In line with many studies [1, 14, 16, 18–20, 22], we found both males and females in our sample overestimated height and there were no significant differences between the sexes [1, 19]. This is consistent with results for China and Russia [21]. There was a tendency to overestimate height among older people, as has been found for China and Russia [21], and this tendency was increased for the older age groups (significantly for those aged 75–79 years and 85 years or older), consistent with other studies [12, 20]. We found age was a significant correlate with height (larger differences with age) and this is consistent with results in the literature [12, 18]. This result could be due to increased postural issues and perhaps cognitive decline associated with psychological distress and the ageing process, as it has been shown that height has been overestimated by more if individuals showed signs of cognitive decline [12]. Also, the larger overestimate may have been related to longer time since height was last measured [1]. In terms of the sexes, females tended to overestimate height by less than males. This would be consistent with osteoporosis leading to increased attention by medical professional on women’s height as osteoporosis has been associated with less overestimation of height by females [12], although we found greater overestimation of height by those with musculoskeletal disorders. Those born in Australia overestimated height by more than immigrants. Differences by cultural groups were also found as there were larger overestimates in height for Caucasians compared to Afro-Caribbean and Asian men in London [38] and larger overestimates of height for Asians compared to Spaniards [34]. This could reflect differences in cultural ideals, as sociocultural factors such as wealth and social norms have been associated with what is considered the desirable body forms in different cultures [21, 34] and therefore body ideals may be culturally bound [39]. Living with others was associated with larger overestimates of height perhaps indicating height perceptions are influenced by consensus or norms (social desirability) [40], although significantly smaller overestimates of height have been found for individuals living with a spouse and others compared to those living alone [12].

Living in inner regional areas was associated with less underestimating of height. This is consistent with results for China that indicated overestimates of height were significantly larger for individuals living in urban areas than rural areas [41]. However, a multi-country study found individuals living in rural areas were likely to overestimate their height by a greater amount than urban residents, attributing the difference to having their height measured more often [42]. Having musculoskeletal disorders was associated with greater underreporting of height and this could be related to cognitive decline (as mentioned earlier), postural issues and spinal compression. In a Norwegian study, Magnusson et al. (2014) showed differences in height were larger for individuals with osteoarthritis compared to those without, but there was insufficient information to determine whether the difference was statistically significant (they did however report significantly greater overreporting of height for individuals who were overweight or obese) [43]. They attributed the effect to social desirability in women and to older people forgetting the probable shrinkage that occurs with age.

Consistent with the literature [1, 14, 20–22], we found older individuals tended to underestimate BMI, and, consistent with other studies [12, 20], our results showed this was increased for older individuals aged 75 or over. This was likely due to the overestimation of height. Modelling results indicated University education was associated with greater underestimation of BMI and was consistent with underestimation of weight. This underestimation of weight was consistent with results for nonmanual workers compared to manual workers [8]. Compared to individuals living in households containing one adult, those living with three or more adults underestimated BMI by significantly more and this is consistent with results for males in [12]. Diabetes was associated with less underreporting of BMI due to the weight result. Hypertension was associated with a larger underestimate of BMI.

### Limitations and extensions

In interpreting the results from our study, it is important to note the limitations. Firstly, our sample consisted of individuals living in the community and excluded those living in non-private dwellings such as residential aged care and hospitals. This omission is important as previous research notes that malnutrition has the highest prevalence among older adults in hospitals and rehabilitation facilities [6]. Secondly, the NHS data were cross-sectional, and we cannot and do not draw causal inferences between the selected demographic and health characteristics and the likelihood of measurement disparities. Finally, although the NHS provided a comprehensive list of variables, a number of factors were not measured that may present as an important correlate of measurement disparities. For example, one study has linked disparities in weight with healthcare visits [44]. Health literacy would also be another important variable. Although the survey contained a battery of health literacy questions, only a (non-representative) subsample of respondents was asked these questions. Omission of these or other exogenous variables may have had implications for confounding factors.

Consistent with some of the literature, our results indicated that the most highly educated individuals had greater underestimation of weight and BMI. This would be somewhat puzzling if education were correlated with health literacy and/or healthcare access leading to greater self-awareness. As the literature did not provide an explanation for this finding, it remains an avenue for further exploration.

## Conclusion

Noting these limitations and potential extensions, the strength of our study lies in the use of nationally representative data with high response rates, to measure the prevalence and correlates of discrepancies in anthropometric measurements and indices among a population of growing pertinence to the healthcare sector. In our study of up to 3412 individuals with plausible anthropometric data aged 65 or over in Australia, we found both males and females underestimated weight (-0.72 kg for males and − 1.26 kg for females), overestimated height (4.05 cm for males and 3.22 cm for females) and underestimated BMI (-1.56 kg/m^2^ for males and − 1.63 kg/m^2^ for females). All else equal, there was evidence that these differences in height and BMI increased significantly with age. There was some evidence that these differences were associated with sex, high levels of education and household composition. Some of these differences could be due to cognitive difficulties associated with the ageing process, more regular access to health practitioners who monitor physical health or social desirability and norms. Although average differences were small, for many individuals the differences were clinically significant, indicating measurements should be taken in clinically focused research and in clinical practice. In addition, these differences could lead to erroneous conclusions on the effects of public health policies and interventions [19]. In particular, systematic underestimation of BMI in older adults can have implications for estimating the older population at risk of related health conditions, such as diabetes, hypertension and functional limitations, and therefore associated impacts on the healthcare system.

## Electronic supplementary material

Below is the link to the electronic supplementary material.

Supplementary Material 1

## Data Availability

The data that support the findings of this study are available from the Australian Bureau of Statistics but restrictions apply to the availability of these data, which were used under license for the current study, and so are not publicly available. Data are however available from the Australian Bureau of Statistics. See: https://www.abs.gov.au/statistics/microdata-tablebuilder/microdatadownload.

## Footnotes

1 https://www.who.int/news-room/fact-sheets/detail/ageing-and-health.

2 For the 54 individuals we dropped, we regarded differences as implausible. For example, differences in weight were mostly about − 40 kg or + 35–50 kg relative to measured weight of 50–150 kg. Height estimates were under or overestimated by as much as 40 cm relative to measured height of 150–190 cm. Most BMI scores were over or underestimated by 10 kg/m2. In our analysis sample, measured weight ranged from 38 to 158 kg, height from 136 to 200 cm and BMI from 16 to 53 kg/m2.

## Abbreviations

BMI: Body Mass Index.
kg: kilograms.
cm: centimetres.
m^2^: metres squared.
NHS: National Health Survey.
