# Supplementary material for: Discrepancies in self-reported and measured anthropometric measurements and indices among older Australians: prevalence and correlates
Source: BMC Public Health. 2022 Oct 17;22:1928. doi: 10.1186/s12889-022-14326-y (PMC9575622; doi:10.1186/s12889-022-14326-y)
Supplement: Supplementary file 1 — Supplementary Material 1 [file 12889_2022_14326_MOESM1_ESM.docx]

## APPENDIX

There were some significant differences between self-reported and measured BMI by education levels (figure A1). Among those with university education, there was a higher prevalence of reported normal BMI (45%) than measured normal BMI (31%) and a lower prevalence of reported obese II (2%) than measured obese II (7%). For those with Diplomas or Certificate III/IV, prevalence of normal BMI reports (28%) exceeded measurements (20%) and obese I measurements (25%) exceeded reports (19%). Among those with the lowest level of education (less than year 12), self reports of normal BMI (30%) exceeded measurements of normal BMI (22%) and actual prevalence of obesity (39%) exceeded measurements of obesity (29%). Those with university education were significantly more likely to report and have had measured normal BMI than the least educated.

|  |
| --- |

Fig. A1. Self-reported and measured BMI by education levels, population weighted.

Among the selected health conditions, those with cardiovascular disease, diabetes and/or hypertension weighed significantly more than the general population of older Australians (figure A2). Interestingly, those with diabetes showed significantly less underestimation of weight than those with other conditions when compared to the population. Arthritis, hypertension and musculoskeletal disorders were associated with less height and cancer with greater height, but there were no significant differences in overreporting of height. BMI results follow the patterns observed in weight.

|  |
| --- |

Fig. A2. Measurements and differences by health condition, weighted.

Note: red line indicates mean for population.

Besides the nominated health conditions examined in figure 3, there were over 100 other health conditions identified in the data. As a measure of morbidity, we investigated differences between self reports and measurements according to the number health conditions (figure A3). However, overlapping confidence intervals indicated no significant differences in the extent of misreporting weight, height or BMI by number of conditions.

|  |
| --- |

Fig. A3. Differences by number of health condition, population weighted.

Note: red line indicates mean for no conditions.

Following on with our analysis of the many detailed health conditions, we examined comorbidities. We took the five most prevalent health conditions in the population of older Australians and examined the top 10 comorbid health conditions, looking for any significant differences in misreporting of weight, height or BMI (figures A4, A5 and A6). We did not see any combination stand out as significantly different from the rest.

| Top 5 conditions, Weight (kg)       |
| --- |

Fig. A4. Differences in weight by main comorbid health condition, population weighted.

| Top 5 conditions, Height (cm)       |
| --- |

Fig. A5. Differences in height by main comorbid health condition, population weighted.

| Top 5 conditions, BMI (kg/m^2^)       |
| --- |

Fig. A6. Differences in BMI by main comorbid health condition, population weighted.

Finally, in our analysis of health-specific factors, we considered a set of variables relating to alcohol risk (short and long term), dietary intake of fruit and vegetables, exercise and physical activity, smoking, area socioeconomic conditions (SEIFA deciles) and Kessler 10 psychological distress categories (figure A7). Among all these variables, there were no statistically significant differences in the extent of weight underreporting, height overreporting or BMI underreporting.

|  |
| --- |

Fig. A7. Differences by main health variable, population weighted.

Note: red line indicates population mean.
